# Editorial: Advances in ruminant viruses

**DOI:** 10.3389/fcimb.2026.1831551

**Published:** 2026-04-13

**Authors:** Muthannan Andavar Ramakrishnan, Santhamani Ramasamy

**Affiliations:** 1 ICAR – Indian Veterinary Research Institute, Bangalore, India; 2 University of Pittsburgh, Pittsburgh, PA, United States

**Keywords:** bluetongue, foot-and-mouth disease, hepacivirus, lumpy skin disease, molecular epidemiology, one health, ruminant viruses, vaccine development

## Introduction

Viral infections in ruminants continue to pose a significant and ongoing threat to livestock health worldwide, impacting food security and agricultural economies worldwide. Although animal virology has greatly enhanced our knowledge of prevention of infectious diseases, research on viruses affecting ruminants has traditionally lagged behind studies of human pathogens. The need to bridge this gap has been emphasized by recent emergence of lumpy skin disease virus causing severe outbreaks in Asia, the resurgence of bluetongue in Europe, and the concerning discovery of highly pathogenic avian influenza A (H5N1) in cattle herds in the United States. These events underscore the urgent requirement for progress in diagnostics, vaccine development, molecular epidemiology, and One Health–oriented surveillance.

The Research Topic titled *“Advances in Ruminant Viruses”* was developed to meet these needs by gathering original research that covers the full scope of viruses affecting ruminant health and production. This includes development of innovative vaccine platforms and studies on pathogenesis to the discovery of viruses and evolutionary genomics. Featured in the *“Veterinary and Zoonotic Infection section of Frontiers in Cellular and Infection Microbiology”*, the Research Topic consists of six original research articles that focuses on virus-host interactions, virus evolution, One Health surveillance and novel vaccine strategy Vaccine Innovation for Foot-and-Mouth Disease.

Foot-and-mouth disease (FMD) continues to be the most economically impactful viral illness affecting cloven-hoofed animals in Asia, Middle East and Africa.Although traditional inactivated vaccines are effective, they face challenges such as the need for cold-chain logistics, high-security production environments, short-term immunity, and the inability to distinguish between infected and vaccinated animals (DIVA). Selvaraj et al. tackle these issues by using a bioinformatics-driven strategy to create thermostable virus-like particles (VLPs) for FMD serotype A (A/IND/40/2000). Through molecular modeling, they identified stabilizing amino acid changes in the VP2 (F62Y) and VP3 (H142D) capsid proteins, resulting in a double-mutant VLP (AM-3) produced in insect cells using the baculovirus system. The AM-3 VLPs showed significantly low degradation (only 62.5% after 15 days at 37 °C) and provided 90% protection in a guinea pig challenge model. This research marks a major advancement towards a next-generation, thermostable, DIVA-compatible FMD vaccine, which holds great practical importance for tropical and resource-constrained regions where cold-chain infrastructure is often lacking.

## Pathogenesis of bluetongue virus serotype 24 in sheep

Bluetongue (BT), a disease caused by the *orbivirus BTV*, is widespread throughout much of the Indian subcontinent and results in significant economic damage. Although various BTV serotypes circulate in India, there is a surprising lack of detailed *in vivo* pathogenesis studies. Vineetha et al. offer the first thorough examination of the pathogenesis and immune responses of BTV serotype 24 (BTV-24) in native Indian sheep. By employing a methodical approach of sequential sacrifice and comprehensive analysis—including gross and histopathology, immunohistochemistry, qPCR-based viral quantification, flow cytometric immune cell profiling, and cytokine measurement—the researchers describe a moderately severe disease progression marked by fever, cyanosis, lymph node and lung pathology, and notable subintimal hemorrhage at the base of the pulmonary artery. Importantly, the persistence of BTV-24 antigen was observed in the tongue, thymus, heart, and pulmonary artery, while a significant increase in pro-inflammatory and antiviral cytokines (IFN-α, IFN-β, IFN-γ, IL-2, IL-12, TNF-α) and apoptotic markers (Bcl-2, caspase-3) was associated with peak viremia. These in-depth pathogenetic findings are crucial for the rational design of vaccines incorporating BTV-24 and for developing evidence-based strategies to control this economically significant serotype.

## Lumpy skin disease: molecular epidemiology and evolutionary genomics

Two complementary investigations by Gundallahalli Bayyappa et al. offer an in-depth molecular overview of the LSD virus (LSDV) within the Indian setting. In their spatiotemporal epidemiological study, Gundallahalli Bayyappa et al. examine 1,353 samples from 451 suspected LSD cases throughout Karnataka (2021–2024), combining PCR-based molecular detection with GIS-enabled spatial analysis tools (QGIS, GeoDa). Their research uncovers significant spatial autocorrelation of cases in densely populated livestock regions, influenced by environmental factors and mechanical vectors such as blood-feeding flies (Stomoxys calcitrans, Haematobia irritans) and ticks (Rhipicephalus and Amblyomma spp.). Notably, scabs (83.6% positivity), nasal swabs (80.9%), and blood (76.9%) proved to be reliable diagnostic materials, with nasal swabs emphasized as a practical, non-invasive option for field monitoring. The risk factor analysis identified cattle aged 1–5 years, females, and exotic breeds (2.1–2.6× higher odds) as the most vulnerable. Positively, vaccination efforts achieving nearly complete coverage in Karnataka reduced LSD-related mortality from 1.45% to negligible levels by 2024, although ongoing hotspots highlight the necessity for continued, region-specific control measures.

In a study on evolutionary genomics, Gundallahalli Bayyappa et al. present the first successful isolation and complete genome sequencing of LSDV from Bos frontalis (mithun), a semi-domesticated bovine species native to Northeast India. Utilizing maximum likelihood phylogenetics, GPCR gene analysis, whole-genome variant calling (identifying 2,212 variants), and haplotype network construction, the researchers show that Indian LSDV isolates form a unique haplogroup, indicating conservative evolution with a relatively recent common ancestor (TMRCA). The study’s findings on transboundary phylogeographic patterns and ongoing genetic diversification offer essential insights for understanding the spread of LSDV and for developing molecular surveillance strategies.

Collectively, these two studies illustrate how combining field epidemiology, molecular diagnostics, spatial analytics, and phylogenomics can enhance our comprehension of an emerging transboundary pathogen and guide evidence-based disease control measures.

## Expanding the host range of hepaciviruses in ruminants

Two research studies conducted in northeastern China have greatly expanded our understanding of the diversity, host range, and potential for cross-species transmission of bovine hepacivirus (BovHepV).

Ma et al. have identified and analyzed BovHepV in cattle and sheep from Hulunbuir, Inner Mongolia. By examining 988 serum samples (468 from cattle and 520 from sheep) across 12 administrative regions using transcriptome sequencing and semi-nested PCR, they discovered six districts with BovHepV presence, showing prevalence rates between 2.0% and 35.0% in cattle and 2.5% in sheep. Phylogenetic analysis placed the strains in genotype 1, subtype G, while recombination analysis highlighted intergenerational links between strains from cattle and sheep. Notably, this research marks the first detection of BovHepV in sheep in northeastern China, confirming cross-species transmission and co-circulation, which has significant implications for understanding hepacivirus ecology and potential zoonotic threats.

Xu et al. expand the known host range by discovering a new genotype of Hepacivirus bovis in reindeer (Rangifer tarandus) from Inner Mongolia. By conducting metagenomic sequencing on 21 reindeer serum samples, they identified two nearly complete hepacivirus genomes (named RtHepV GH01 and GH02), with GH01 showing a 42.9% positivity rate. Phylogenetic analysis positioned these variants within the Hepacivirus bovis lineage but in a separate clade, while p-distance analysis verified them as a new genotype rather than a different species. The presence of recombination between RtHepV and BovHepV strains, along with co-evolutionary studies indicating frequent host-switching events within the *Hepacivirus* genus, highlights the exceptional ecological adaptability of these viruses and the persistent risk of cross-species transmission.

## Synthesis and future directions

The six articles featured in this Research Topic collectively push the boundaries of ruminant virology in various ways. Several overarching themes are evident:

1. Translational vaccine innovation: The thermostable VLP platform for FMD (Selvaraj et al.) exemplifies the effectiveness of computational biology in addressing persistent challenges in vaccine distribution within resource-constrained environments, a model that can be extended to other ruminant diseases.
2. Detailed pathogenesis for informed control: The BTV-24 research (Vineetha et al.) addresses a significant gap in knowledge, offering the immunological and pathological foundation essential for developing rational multiserotype vaccines in regions where BTV is prevalent.
3. Integrative molecular epidemiology: The complementary LSD investigations (Gundallahalli Bayyappa et al.) illustrate how the integration of field epidemiology, GIS-based spatial analysis, and whole-genome phylogenomics can inform targeted surveillance and intervention strategies for transboundary pathogens. Further, this study provides a framework for understanding the evolutionary trajectory of animal viruses.
4. Viral discovery and One Health surveillance: The hepacivirus research (Ma et al.; Xu et al.) broadens the recognized host range and geographical spread of Hepacivirus bovis to include sheep and reindeer, highlighting active cross-species transmission and emphasizing the importance of One Health–oriented viral monitoring at the interface of livestock and wildlife.

Looking forward, the results highlighted here suggest several key focus areas: (i) progressing thermostable vaccine platforms to create field-ready products for FMD and other viral diseases affecting ruminants; (ii) conducting systematic studies on the pathogenesis of circulating BTV serotypes to guide the development of polyvalent vaccines; (iii) establishing real-time, genomics-based surveillance networks for LSDV and other transboundary pathogens; and (iv) enhancing metagenomic monitoring of hepaciviruses and other emerging viruses at the intersection of livestock, wildlife, and humans.

Overall, this Research Topic highlights the following: The computational and bioinformatic tools are invaluable for understanding virus biology, fitness, and evolution, as well as for advancing translational research such as vaccine development. This underscores the necessity of collaboration across disciplines. Insights into virus-host interactions can inform effective interventions to address emerging and re-emerging virus threats. The rapid evolution of viruses, particularly RNA viruses, may facilitate inter-species spillover (e.g., influenza virus H5N1), highlighting the need for One Health surveillance at the human-animal interface.

We extend our gratitude to all the authors for their diligent and influential research, to the reviewers for their insightful feedback, and to the editorial team at Frontiers in Cellular and Infection Microbiology for their assistance in realizing this Research Topic. We hope this compilation will be a significant asset to the scientific community and will inspire further progress in the understanding, prevention, and management of viral diseases in ruminants.

